# The European Certificate in Medical Genetics and Genomics (ECMGG)

**DOI:** 10.1038/s41431-025-01889-8

**Published:** 2025-07-02

**Authors:** Peter D. Turnpenny, Laura Pölsler, Ute Moog, Edward S. Tobias, Angela Peron, Susanne E. Boonen, Bonnie Lynch, Jonathan Berg

**Affiliations:** 1 University of Exeter Medical School, Exeter, UK; 2 Centre for Medical Genetics, Clinical Sciences, Research group Genetics, Reproduction and Development, Vrije Universiteit Brussel (VUB), Brussels, Belgium; 3 Institute of Human Genetics, Heidelberg University, Heidelberg, Germany; 4 Queen Elizabeth University Hospital, University of Glasgow, Glasgow, Scotland UK; 5 Division of Medical Genetics, Meyer Children’s Hospital IRCCS, Florence, Italy; 6 Department of Experimental and Clinical Biomedical Sciences “Mario Serio”, Università degli Studi di Firenze, Florence, Italy; 7 Department of Clinical Genetics, Odense University Hospital, Odense, Denmark; 8 Centre for Medical Education, University of Dundee, Dundee, Scotland UK; 9 Ninewells Hospital and Medical School, University of Dundee, Dundee, Scotland UK

**Keywords:** Medical genetics, Health care

## Abstract

The European Certificate in Medical Genetics and Genomics (ECMGG) is the official knowledge-based, end-of-specialist training examination designed and delivered by the Union Européenne des Médecins Spécialistes – Section of Medical Genetics (UEMS-SMG). The examination is a joint venture of the SMG, the European Society of Human Genetics (ESHG), and the European Board of Medical Genetics (EBMG). Sittings have taken place in 2019 and 2021–24, and it is gaining in reputation as a high-quality, high-standard assessment. In 2024 the ECMGG underwent satisfactory appraisal by the UEMS-Council of European Specialist Medical Assessment (CESMA). This paper describes the development of the ECMGG, its structure, outcomes, and its meaning for the standards and harmonisation of the specialty of Medical Genetics throughout Europe.

## Introduction

The UEMS (https://www.uems.eu/) is a non-profit organisation based in Brussels and founded in 1958, with UEMS Specialist Sections first created in 1962. There are now 43 Specialist Sections and a number of Multidisciplinary Joint Committees and Thematic Federations. In addition, cross-disciplinary Working Groups, including those focussed on Continuing Medical Education and Postgraduate Training, are active. Membership comprises 41 *full* member nations, 6 *associate* members, and 4 *observer* nations. The overarching goal of the UEMS is, *“…to improve patient care throughout Europe by developing and supporting excellence in specialist medical practice.”* To this end the UEMS specialist bodies concentrate activity on raising standards of training and harmonising training programmes across Europe, principally through producing European Training Requirements (ETR) and constructing an end-of-training examination for their specialty. In addition, appraisal of training centres across Europe is underway for some specialties. The first European examination, in anaesthesiology, was organised in 1984, and to date more than 30 specialties have exams.

**Fig. 1:**
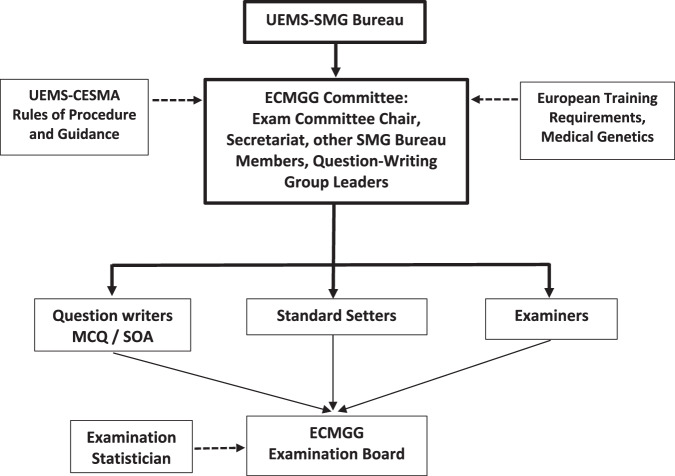
ECMGG Examination Group organisation and governance structure.

The Section of Medical Genetics (SMG; https://uems-genetics.org/) was established in 2013. The SMG’s first ETR was completed and approved in 2017, and a full revision approved in 2023 [1]. Work began on the ECMGG in 2017 as a joint venture with the European Board of Medical Genetics (EBMG) and the European Society of Human Genetics (ESHG), though fully undertaken by the SMG Examination Group (Fig. 1). It is primarily a knowledge-based exam for trainees in medical/clinical genetics in the final phase of their training prior to being appointed as senior doctors/consultants. It aspires to help establish European specialty standards to world class levels and incorporates an assessment of clinical competencies through the oral component, acknowledging this does not substitute for the ongoing appraisal of a trainee’s competencies over the entire training period with workplace-based assessment. The exam is only available to medically trained geneticists, is delivered in English only, and there is no limit to the number of times a candidate may sit the exam.

Across Europe, the specialty of medical or clinical genetics varies considerably, the main divide being the difference between largely patient-facing practice and largely laboratory-based practice. The ECMGG focusses on clinicians who have a patient-facing practice, but includes the required laboratory knowledge for this role. Clinicians who are purely laboratory based have the option of accreditation and examination through the EBMG branch of Clinical Laboratory Genetics.

Differences in clinical practice between countries remain a challenge. The ethos of the exam is that it adheres to the ETR. The consensus question-writing process ensures that each MCQ and SOA question is reviewed by geneticists from several European countries, avoiding highly specific national practice. Candidates should also appreciate the variation in practice across Europe, but specific requirements of national protocols are not tested.

The choice of administering the exam only in English is the most common option for exams administered by the UEMS. Although sub-optimal, this is because of the broad usage of English in medical education, with the likelihood that a candidate will have competency in English as a second language. The logistics of having different language options are too problematic, as the exam would have to be translated and then standard set separately. Each additional language would then only make it more accessible to a small proportion of candidates.

The exam was designed with a view to making it appropriate and acceptable as an ‘exit exam’ for training in the specialty of Clinical Genetics. How and when the exam is used as a training requirement is, however, a matter of acceptance by the medical associations of individual countries. To date, 3 countries have accepted the exam as a formal specialty assessment.

The exam (initially EDMGG: the European Diploma in Medical Genetics and Genomics; https://www.uems-ecmgg.org) was developed through twice-yearly, in-person workshops from October 2017 to March 2020, with 8–12 attendees. It has two parts: (1) a multiple-choice question (MCQ) paper, and (2) an oral exam in the form of a Structured Oral Assessment (SOA) consisting of OSCE (Objective Structured Clinical Examination)-style stations. A pilot exam was delivered in June 2018, with 8 candidates answering 60 MCQs and rotating through 3 SOA stations.

The first official exam took place in Gothenburg, 2019, in hard-copy format with 100 MCQs and 6 SOA stations. Candidates had to pass both components of the exam, with no compensation for lower grades between exam components. The pass mark was determined by accepted standard setting methodologies, Angoff for the MCQ and Hofstee for the SOA exam. Twenty candidates sat both parts of the exam and 15 (75%) passed. Psychometric analysis was performed by Dr Bonnie Lynch, University of Dundee exam statistician. The exam was suspended in 2020 due to the Covid-19 pandemic with a decision to develop it fully online for 2021, renaming it the ECMGG. CYIM (Cyber Imagination; https://www.cyim.com/en), based in Rennes, France, was chosen as the delivery partner to provide the proctored exam platform and, in 2022, an online database for managing MCQs.

The first fully online ECMGG was delivered in June (MCQ part) and September (SOA part), 2021. The MCQ paper was expanded to 110 questions to allow for removal of a small number of poorly performing questions following psychometric analysis. The SOA remains as 6 OSCE-style stations, now delivered using Zoom. In 2023 and 2024 the schedule was brought forward to April (MCQ part) and June (SOA part). The annual schedule is shown in Table 1.

**Table 1:** The annual schedule for preparing and delivering the ECMGG.

September	MCQ writing in small groups online
**Late September**	1. Update Examination General Document 2. Update ECMGG websites *re* next year’s exam
**From October 1**^**st**^	Candidate registration opens
**November**	First SOA question writing workshop online
**November—December**	Finalise 120 MCQ draft paper
**Late December**	Draft MCQ paper distributed to standard setters
**January—February**	1. Standard setters assess draft MCQ paper 2. Continue preparation SOA questions
**31**^**st**^ **January**	Candidate registration closes
**February**	Candidate exam fees received
**March**	1. In-person question-writing and exam business workshop 2. Finalise 110 MCQ paper
**Early April**	Candidate briefing sessions for MCQ paper
**Late April**	MCQ Exam
**Early May**	1. Review MCQ paper after statistical analysis, remove poorly performing questions 2. Revise statistical analysis MCQ paper
**Mid-May**	1. Convene virtual Interim Examination Board 2. Deliver results of MCQ exam to candidates
**Late May**	Finalise SOA questions
**Early-Mid June**	Examiner and candidate briefing for Oral exam
**Mid-Late June**	Oral exam
**Late June**	1. Review outcome Oral exam 2. Convene Final Examination Board, followed by Examination Committee meeting
**Early July**	Deliver ECMGG results to candidates
**Late July**	1. Give feedback to candidates as necessary 2. Deliver ECMGG certificates to candidates

In-person workshops resumed in 2022 on an annual basis and now include a training day to improve question-setting and examining skills. All other exam preparation is conducted virtually. The ECMGG was appraised in 2024, and given 5 years full accreditation by the Council of European Specialist Medical Assessment (CESMA; https://www.uems.eu/cesma-appraisals), the UEMS body for governance oversight of European examinations.

## ECMGG design and structure

The content of the exam is based on the syllabus of the ETR for the specialty of Medical Genetics (https://drive.google.com/file/d/1pMuUQTGRPdXxBYH4uvQeku77NhOCvSpH/view). The breadth of content is matched to a simplified list of topics and domains (Table 2). A structured blueprint for the exam has been developed recently and is being introduced from 2025.

**Table 2:** Simplified topics and domains covering the ECMGG syllabus.

**Topic / Sub-specialty Codes**	
A	Basic including lab methods
B	Metabolic
C	Malformations
D	Intellectual disability and autism
E	Neurogenetics + neuromuscular + psychiatric
F	Cancer
G	Cardiac
H	Skeletal + connective tissue disease + multi-systemic vascular disease
I	Ophthalmologic
J	Endocrine
K	Gastrointestinal and hepatic
L	Haematologic + immunological + autoimmune
M	Dermatologic
N	Renal and urogenital
O	Pulmonary
P	Gynaecological and obstetric disease and infertility
Q	Craniofacial + Ear-Nose-Throat
**Domain Codes**	
a	Clinical diagnosis, dysmorphology – investigations, including metabolic tests
b	Management, therapy
c	Prenatal / reproductive + embryology
d	Inheritance, pedigree, risk assessment
e	Epidemiology and population, screening, pharmacogenomics
f	Molecular genetics, including epigenetics
g	Cytogenetics
h	Multifactorial
i	Counselling and communication
j	Ethical, Legal and Social Implications
k	Mechanisms
l	Bioinformatics

All MCQs are catalogued with up to 3 topics and up to 3 domains.

### MCQs

MCQs in the paper follow the ‘best answer of 5’ format, candidates selecting the best answer from 5 options. This complies with UEMS-CESMA guidance outlined in, ‘A Guide to Successfully Writing MCQs’ (Type A question; https://drive.google.com/file/d/1a17KFLcpNn7loJMho39KFB1BlsS3s-Fe/view). It is also compliant with the internationally recognised format used for the MRCP(UK) Part 1 and 2 exams (available on request from the Federation of the Royal Colleges of Physicians of the UK; https://www.thefederation.uk/). Questions contain: (1) a clinical vignette which is brief, simple, and sets the context; (2) data or test results (optional); (3) a lead-in question; (4) five possible answers. A consistent style is followed with the English as simple as possible. Non-standard abbreviations are avoided, and many well-known abbreviations are expanded. The data or test results are kept to a minimum. Genetic testing results follow HGVS nomenclature where appropriate. Other data provided may include a radiograph, clinical image, or a pedigree, in addition to any other appropriate clinical test; normal ranges are provided where relevant. As is standard for MCQ writing, in both the lead-in question and answer options, ‘negatives’ are avoided. The answer options for each question should be short, roughly the same length, and in alphabetical or numerical order. They should all be of same type, e.g. ‘genetic investigations’ or ‘organ systems’, and ‘single’, i.e. non-combinatorial. In addition, great care is taken to avoid any ambiguity.

Candidates have 2 h 45 m to answer 110 MCQs, i.e. 90 s per question. Questions can be answered in any order and there is no penalty for a wrong answer. It is a ‘closed book exam’ and the candidate’s environment is proctored, with no mobile phones, devices, or writing materials permitted. This limits the ability to set questions involving complex calculation, for example around Bayes’ theorem.

### SOAs

Since 2021 only candidates who pass the MCQ paper advance to the oral exam. Candidates rotate through six 10-min OSCE-style stations. A small team write and review the SOA scenarios, using a framework described below.

The stations cover broad areas of clinical practice with the exam conducted in English. Allowance is made for different English language ability levels as far as possible. At each station the candidate is shown a single screen presenting a clinical scenario, often with a pedigree and/or genetic test results. The candidate reads the page for 2 min, following which the examiners ask 3 questions over 8 min (not visible on screen). The questions cover three domains: (1) Application of Genetic Principles; (2) Clinical Communication and Counselling Skills; and (3) Clinical, Ethical, and Legal Aspects. Detail of the current framework is shown in Table 3.

**Table 3:** Framework for the SOA exam.

Application of genetic principals	
A	Penetrance and variable expression
B	Variant classification and interpretation in the clinical setting
C	Anticipation and triplet repeat diseases
D	Risk assessment, epidemiology, population genetics and multifactorial
E	Difficult inheritance: mitochondrial inheritance, imprinting effects
F	Incidental findings
G	Interpretation of pedigree information
H	Limitations of specific testing methods
I	Genetic mechanisms: dominant negative effects, two-hit hypothesis, oncogenes
J	Syndromic vs. Non-syndromic symptoms – stepwise clinical evaluation
Clinical, Ethical and Legal aspects	
a	Prenatal testing issues
b	Preimplantation diagnostics (PGT)
c	Predictive testing in children
d	Predictive testing in adults
e	Commercial testing
f	Community testing
g	GDPR and confidentiality
h	Financial aspects and insurance related topics
i	Future implications
j	Informed consent
k	Treatment of genetic diseases
Clinical communication and counselling skills	
I	Communication with the general practitioner
II	Communication with another specialist
III	Communication with patients
IV	Communication with parents
V	Communication with siblings
VI	Communication with distant relatives
VII	Communication with (adult) children
VIII	Communication with laboratory

For each question, 1 topic is selected from each of the 3 domains.

Each station has 2 examiners (sometimes a third *in reserve*, or observing), who may be external examiners or members of the Examination Group. One examiner questions the candidate while the other takes notes, with these roles alternating. Both examiners independently score candidates in each of the 3 domains: **4** – Clear Pass; **3** – Borderline Pass; **2** – Borderline Fail; and **1** – Clear Fail. Over 6 stations this process generates 36 data points for each candidate.

## Validation and analysis

The exam is designed to identify a ‘minimum passing candidate’ – someone who has just the level of knowledge for safe clinical practice in a senior role.

### MCQs

**Fig. 2:**
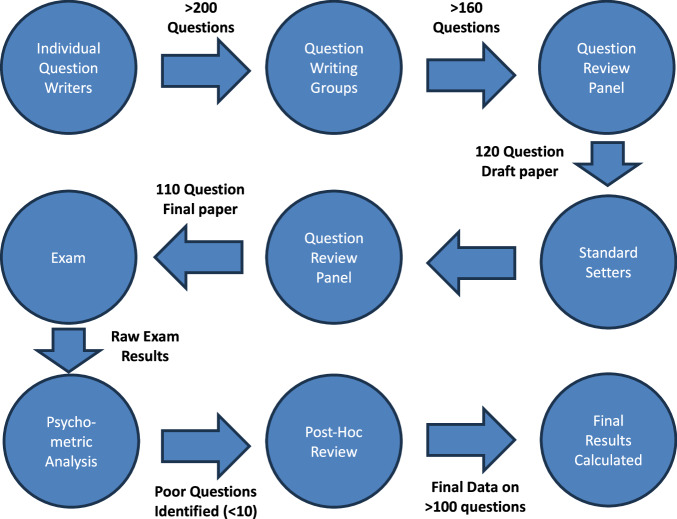
Over 200 questions are written by individual question writers. These are reviewed and edited ‘live’ in question writing groups of 4–6 people. Around 160 questions are then reviewed by a review panel of at least 3 experienced geneticists, and the 120 question draft paper is assembled. This paper is sent to standard-setters, whose comments are reviewed with the questions, and a final 110 MCQ paper is constructed and checked against the blueprint. After the exam, psychometric analysis is performed and poorly performing questions are reviewed. Questions which have a clear reason for performing poorly are removed. The final marks and passes are calculated after removing these questions from the analysis. Each question that is used has been reviewed at 3 different stages, including more than 20 people.

All MCQs undergo a review process outlined in Fig. 2. Briefly, each question submitted by a question writer is reviewed and edited in a small group, followed by a second-line review by at least 3 experienced examiners before a draft paper of 120 questions is assembled and sent for standard setting.

Standard setting uses the Angoff method [2] with assessment by at least 15 experienced clinical geneticists who have not written the questions. Comments may be submitted by standard setters, and at the final paper review 10 MCQs are removed, leaving a 110-question paper. The simple Angoff method takes the average of all the standard setters’ estimates for each of the 110 original items, with a grand mean of all item averages then computed to calculate a passmark/cut score. No correction/modification is used.

After the exam, question performance is assessed using split-half reliability, Cronbach’s alpha, item validity, and item difficulty.

*Split-half reliability* calculates how similar the candidates’ performance is in the first, versus the second, half of the exam. A reliability score of 0.80 or above is widely accepted as desirable, suggesting that candidates are not becoming fatigued by too many questions.

*Cronbach’s alpha* assesses the overall measure of the exam’s reliability, or *internal consistency*. The performance on each item is correlated with performance on the overall exam, and, with inter-item correlations calculated for each item to permit an item-level analysis of the exam’s reliability. For Cronbach’s alpha, there is no consensus on a minimum acceptable value; 0.70 or greater is considered adequate by some, while 0.80 is the accepted threshold for others. Briggs and Cheek (1986) [3] suggested that Cronbach’s alpha is too sensitive to the number of items, thereby representing a potentially inflated marker of internal consistency. For this reason, they recommend using the mean of the raw item-total correlations as the measure of internal consistency.

The *item analysis*, or *validity*, is the extent to which items measure what they are intended to measure. Items that were answered incorrectly by ≥80% are reviewed, in addition to items with a low or negative discrimination. A small review panel assesses each poorly performing question. Common issues include a question with 2 correct answers, or a confusing question with poor or ambiguous phraseology. Questions are removed if a valid reason is identified. In 2024, item discrimination values ranged from −0.27 to 0.67, with an average value of 0.27, which is a slight decrease relative to the 2021, 2022 and 2023 values of 0.33, 0.37 and 0.30, respectively.

Following removal of poorly performing questions, candidate scores and the cut score are recalculated and passing candidates identified.

*Item difficulty* is denoted as the percentage of examinees who answer an item incorrectly. In each year the exam has demonstrated a balance of easy, moderate, and difficult content in almost equal proportions.

Following analysis of the MCQ, an Interim Examination Board convenes online to discuss and ratify the results.

### SOAs

The SOA scenarios and questions are rigorously reviewed for content and language. Concerning *reliability*, because examiners do not share an adequate number of candidates to allow for calculation of the intraclass correlation coefficient or a simple Spearman correlation assessment, inter-rater reliability is not possible.

The Hofstee method [4] is used to set the pass mark, with the following parameters established by the expert panel: lowest acceptable fail rate = 0%; highest acceptable fail rate = 25%; lowest acceptable pass mark = 72.2%; highest acceptable pass mark = 75%. The resulting pass mark was 73.5%. An additional criterion of having a mean raw score ≥2 in 5 of the 6 stations was set.

Whereas the distribution of MCQ scores approximates to a normal distribution, the distribution of SOA scores is skewed toward higher scores, partially explained by the fact that candidates who sat the SOA had already passed the MCQ paper and represent a high-performing cohort. (Data shown in supplementary Fig. A, B).

The Hofstee standard setting graph consistently shows intersection of the H line within the area defined by the parameters we use, indicating reliability.

Following analysis, a Final Examination Board convenes online to ratify the anonymised results. For both Interim and Final Board meetings anyone from the extended Examination Group may attend.

## The Examination Group and demographics

The Examination Group in its entirety comprises question writers, standard-setters and examiners (Fig. 1). There is no overlap between question writers and standard-setters but examiners for the Oral exam may come from either of these groups or none. Examiners must be from countries whose national specialist organisation is a member of the UEMS-SMG, with a minimum of 5 years’ experience at senior level. Diversity of geographic origin across Europe is sought, acknowledging there is significant national variation in experience with medical genetics training. Attempts are being made to reduce the predominance of examiners from northwestern European countries. The demographics of the Examination Group is shown in Table 4.

**Table 4:** Demographics of the Examination Group, broken down into Question Writers / Examination Committee (2024 – present membership), Standard Setters (2022-24), and Examiners (2022-24).

Nation	Exam Committee / Question Writers (2024)	Standard Setters (2022–24)			Examiners (2022–24)		
		2022 (*n* = 17)	2023 (*n* = 15)	2024 (*n* = 17)	2022 (*n* = 24)	2023 (*n* = 21)	2024 (*n* = 25)
Belgium	1				1	2	1
Denmark	2	1	1	1	1	1	2
Finland					1	2	1
France	1				1	1	1
Germany	3	5	6	6	6	2	6
Greece	1						
Hungary					1	1	
Italy	1					1	
Malta	1				1	1	1
Netherlands		2	1	1			1
Norway	1	1	1	1			
Romania	1			1	1	1	1
Slovenia		1	1	1	1		1
Spain	2				1	1	1
Sweden	2	1	1	1	2	2	1
Switzerland		1	1	1			
Turkey	1				2	1	1
UK	3	5	3	4	5	5	7

The pool of Examiners is larger; the ‘Examiners’ numbers do not include colleagues present online in an administrative role or observing in order to prepare to be an Examiner in the future.

## Candidate demographics and background

The ECMGG is an exam designed to align with European training and European standards. Thus, the majority of candidates will be training and/or practising in European centres. The exam has also proved attractive for trainees in countries with *associate* UEMS membership, mainly Turkey, as well as the rest of the world. Table 5a, b show the demographics for all exams. A total of 219 candidates have registered for the ECMGG (including EDMGG in 2019), with 58% from *full* UEMS member nations, 22% from *associate* member nations, and 20% from the rest of the world. Some candidates are established senior doctors rather than trainees.

**Table 5:** a Demographics of the Candidates, 2019 and 2021–24. b Additional demographics for ECMGG 2024 candidates.

a				
Year	Total no. Registered Candidates	Europe – UEMS Full Members *N* (%)	Europe – UEMS Associate Members^a^ *N* (%)	Rest of the World *N* (%)
**2019**	21^b^	15 (71.4)	4 (19)	2 (9.5)
**2021**	38	21 (55.3)	12 (31.6)	5 (13.1)
**2022**	58^c^	31 (53.5)	13 (22.4)	14 (24.1)
**2023**	42^d^	28 (66.6)	9 (21.4)	5 (12)
**2024**	60^e^	32 (53.3)	10 (16.7)	18 (30)
**TOTALS**	**219**	**127 (58)**	**48 (22)**	**44 (20)**

b					
Exam	Male	Female	UEMS Full Members	UEMS Associate Members	Rest of the world
**MCQ**^f^	20	40	32	10	18
**SOA**	8	17	17	4	4

^a^In all but 4 cases (2 Georgia, 1 Serbia, 1 Ukraine) these candidates were from Turkey.

^b^One candidate failed to attend the in-person exam.

^c^One candidate, from an Associate nation, did not attend due to a personal error with time zones.

^d^Three of the 2023 candidates did not sit the exam because of software compatibility problems.

^e^Four of the 2024 candidates did not sit the exam, two because of failed internet connections, two due to personal errors with time zones.

^f^Four of the 60 candidates who registered in 2024 did not sit the exam, two because of failed internet connections, two due to personal errors with time zones.

For various reasons, given in Table 5a, 9/219 candidates (4.1%) either failed to attend, misunderstood their time zone for joining the online exam, or experienced internet connection problems. Table 5b shows additional demographics for 2024, requested for the CESMA Appraisal.

## Examination outcomes

Table 6 describes the outcome data available for all exams since 2019. The overall proportion of passing candidates is just above 50%, ranging from 75% in 2019 to 41.1% in 2024. In 2024 ‘rest of the world’ candidates comprised 30%, and for 2021–24 the pass rate for *full* UEMS member nations has been ~55–60%. In relation to pass rate, there appears to be a broad correlation according to whether candidates have undertaken structured training in established centres.

**Table 6:** ECMGG Examination outcomes.

Year	Number of Candidates Sitting Exam	Passmarks		Number Passing MCQ Paper and Proceeding to Oral Exam *N* (%)	Number Failing Oral Exam *N* (%)	Overall Passes *N* (%)
		MCQ	Oral			
**2019**^a^	20	71%	60%	N/A	N/A	15 (75)
**2021**	38	66.85%	73.70%	21 (55.3)	1	20 (52.6)
**2022**	57	68.36%	72.20%	32 (56.1)	3	29 (50.9)
**2023**	39	71.43%	73.50%	23 (59)	2	21 (53.8)
**2024**	56	70.72%	73.50%	25 (44.6)	2	23 (41.1)
**TOTALS**^a^	**210**	**69.34% Avge (2021–24)**	**73.22% Avge (2021–24)**	**101 (53.2) (2021–24)**	**8 (7.9) (2021–24)**	**108 (51.4)**

^a^The candidate numbers and overall passes for 2019 have been included in the totals but the remainder of the data under ‘Totals’ relates to the years 2021–24, i.e. the years of the online ECMGG.

There is a concern that administering the exam in English risks discriminating individuals on linguistic grounds. Candidates have the opportunity to feedback after each exam. Although the number of candidates completing feedback has decreased, only 2/10 candidates in 2023 stated that they had difficulty with the language, with no such statement in other years.

Most candidates who pass the MCQ paper pass the oral exam, and from 2021–24 between one and three candidates have not passed it, given in Table 6. The reasons have not been analysed but in some cases may relate to candidates being more laboratory-based rather than clinic-based in their practice. Feedback is available to candidates after the Oral exam, if requested, based on examiners’ documentation. Currently, feedback other than overall pass mark is not available after the MCQ exam.

## Security

Everyone involved in writing, standard setting, examining, or administrating the exam is required to sign a Declaration of Confidentiality document, relating particularly to the questions and content. This is renewed every three years.

Candidates are required to present their ID at both parts of the exam. The platform provider, CYIM, links with ProctorU (https://www.proctoru.com/), an online provider. Thus, connection to the MCQ paper involves both a link provided by CYIM and another to ProctorU. In this system, one proctor will watch four candidates during the course of the exam, checking they do not consult any sources of information relating to the questions. The ProctorU connection will not tolerate any rogue or remote access software on the candidate’s computer. Candidate anonymity is preserved during both the Interim Board and Final Board meetings.

The SOA exam has required three rounds to process all candidates, raising the potential for candidates who have completed the exam in the first round to communicate with candidates waiting to join for the third round (a point raised by the CESMA Appraisal). This risk is minimised by grouping candidates according to their work locations. There are differing opinions as to how much communication across groups in OSCE-style exams presents a risk to exam integrity.

## CESMA appraisal

CESMA aims to appraise all exams every 5 years. An application was submitted in October 2023 for the ECMGG 2024. Two appointed appraisers joined the various online meetings in both the preparation phase and the delivery of the exam. The outcome was very positive, praising a high-quality, well-organised exam with good communication to candidates. The lack of insurance cover was highlighted as the main issue needing to be addressed. The exam was validated for 5 years.

## Discussion

The project to develop a European end-of-training exam for medical geneticists, aimed at both a European and world-leading standard, is entirely consistent with the overarching goal of the UEMS: *“…to improve patient care throughout Europe by developing and supporting excellence in specialist medical practice.”*

Developing and supporting excellence in the specialist practice of medical genetics can be conducted along several lines, including high quality research and technical innovation (showcased annually at the ESHG Conference), pursuing educational and training initiatives through specialised courses and workshops, and the growth of training opportunities in expert training centres [5]. Whilst medical genetics services are multi- and inter-professional (including clinical laboratory geneticists and genetic counsellors), the UEMS-SMG, by necessity, is invested in raising standards for medically trained geneticists. It has sought to do this by establishing, through consensus, the European Training Requirements and syllabus [1], and a high-quality, high-standard end-of-training exam, the ECMGG. Those who work towards this qualification will seek to bring their knowledge base to the required level and demonstrate clinical competence in the oral exam.

The ECMGG is therefore a significant tool in the armoury of training for both harmonising and raising standards. There is significant diversity across European nations in both the size and structure of medical genetics services [6] as well as training opportunities for the next generations of specialists. Improving the situation requires a long-term, ongoing effort, and to this end a large team of volunteers, in most cases devoting many hours of their own time, has coalesced around the ECMGG project. Those who contribute now come from the whole generational spectrum of medical geneticists across Europe, i.e. those relatively recently appointed to senior doctor or consultant positions as well as those with decades of experience. Only now, at the time of writing, has some salaried part-time administrative assistance been recruited, courtesy of the European Reference Network-Ithaca (https://ern-ithaca.eu/).

## Outlook

The ECMGG does not possess any intrinsic authority as a qualification, and to do so requires official recognition by the regulatory training authorities of nation states. To date it is recognised in Belgium, Ireland and Malta, is being introduced concomitantly with national exams in Finland and Slovenia, is being considered by the United Kingdom, and is *recommended* in other countries, e.g. Sweden.

We have not formally studied barriers to, or enablers of, national adoption. Some countries perceive their current training process as sufficient, not requiring the considerable effort of changing the system. In other countries the robust design of the ECMGG exam, a large group of examiners and a large enough cohort of candidates to allow formal psychometrics are positive factors that have supported adoption, or may lead to adoption in the future. In countries where the exam has been formally adopted, this has been considerably assisted by the advocacy of the national UEMS representatives.

There is a risk that the ECMGG continues to reinforce the trend of a divide between clinically-based and laboratory-based genetic training. Although this could be mitigated by the option of taking both the ECMGG and the EBMG Clinical Laboratory Genetics exam. In the longer term, reducing the divide requires better harmonisation of national training requirements and medical roles across Europe.

The long-term success of the ECMGG project will likely be measured according to its increasing adoption by European national medical training regulatory bodies as a recognised end-of-training examination. All the indications are that this is gaining momentum and many young candidates, in numerous countries, are sitting the exam even though it is not a mandatory requirement for them. To develop the exam further, we offer our most successful candidates the opportunity to participate in the exam group, ensuring that the most up to date knowledge of the specialty is represented.

With increasing importance of the exam, there will be a need to strengthen the support for candidates, in terms of local mentorship as well as provision of courses. As more individuals pass the exam from different countries, we hope that this will provide some of the mentorship required. Input from national UEMS section representatives already contributes to promoting and supporting the exam in different nations. Courses that are directed at the European Training Requirement and the ECMGG will be required, and could be organised collaboratively with the ESHG, providing an independent and experienced organisation for such courses.

The years 2019–24 has been a period of developing and establishing the ECMGG. The next phase is to progress the project to ‘business as usual’ with its schedule and processes (Table 1) established as routine and robust.

## Supplementary information

Supplementary figures

## Data Availability

Minutes of the Examination Committee and Examination Reports are available on the SMG website (https://uems-genetics.org/). Minutes of the Interim and Final Examination Board meetings are archived in a password-protected area of the same website, as is the CESMA Appraisal report for 2024.
