# Supplementary material for: The European Certificate in Medical Genetics and Genomics (ECMGG)
Source: Eur J Hum Genet. 2025 Jul 2;33(9):1113–20. doi: 10.1038/s41431-025-01889-8 (PMC12402500; doi:10.1038/s41431-025-01889-8)

Supplementary Figure A

Score distribution, ECMGG MCQ Examination 2024, expressed as percentages; 56 candidates. The black curve represents a normal distribution. The graphic suggests some skew toward higher scores but neither skewness nor kurtosis statistics approached significance, indicating that for analysis purposes, the data could be considered normally distributed.

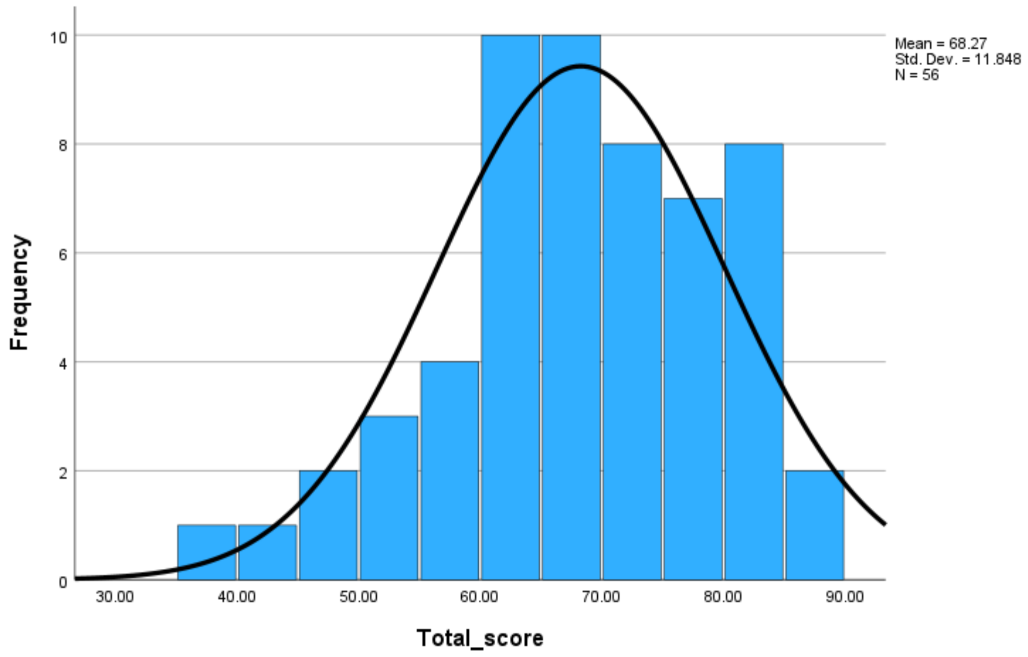

Supplementary Figure B.

Score distribution, ECMGG Oral examination 2024, 25 candidates.

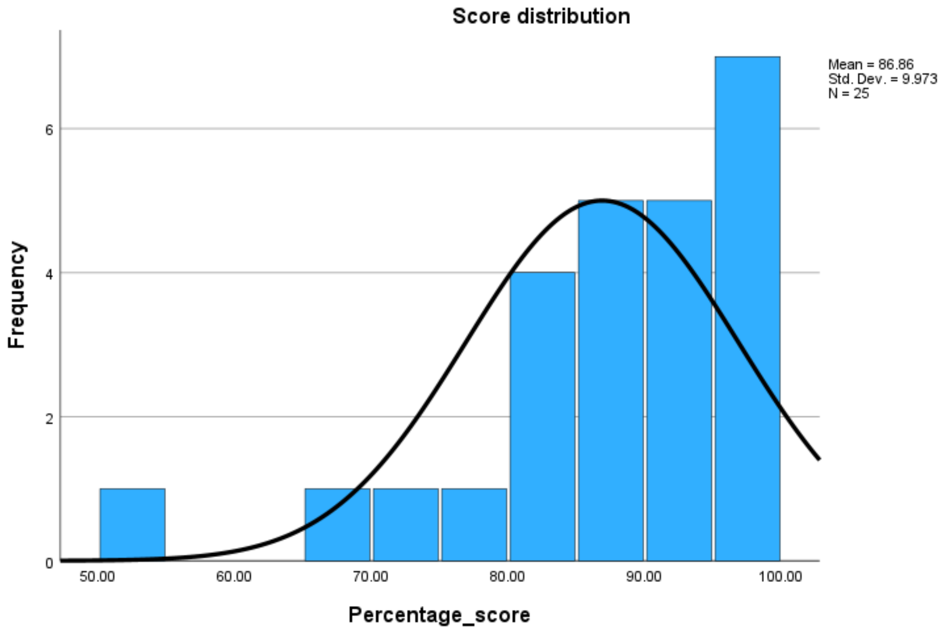

Supplement: Supplementary file 1 — Supplementary figures [file 41431_2025_1889_MOESM1_ESM.pdf]
